# Terminal Restriction Fragment Length Polymorphism for the Identification of Spirorchiid Ova in Tissues from the Green Sea Turtle, *Chelonia mydas*

**DOI:** 10.1371/journal.pone.0162114

**Published:** 2016-08-31

**Authors:** Phoebe A. Chapman, Rebecca J. Traub, Myat T. Kyaw-Tanner, Helen Owen, Mark Flint, Thomas H. Cribb, Paul C. Mills

**Affiliations:** 1 Veterinary-Marine Animal Research, Teaching and Investigation Unit, School of Veterinary Science, The University of Queensland, Gatton, Queensland, Australia; 2 Faculty of Veterinary and Agricultural Sciences, University of Melbourne, Parkville, Victoria, Australia; 3 School of Forest Resources and Conservation, University of Florida, The Florida Aquarium’s Center for Conservation, Apollo Beach, Florida, United States of America; 4 School of Biological Science, The University of Queensland, St Lucia, Queensland, Australia; University of Minnesota, UNITED STATES

## Abstract

Blood flukes are among the most common disease causing pathogens infecting vertebrates, including humans and some of the world's most globally endangered fauna. Spirorchiid blood flukes are parasites of marine turtles, and are associated with pathology, strandings and mortalities worldwide. Their ova embolize in tissues and incite significant inflammatory responses, however attempts to draw correlations between species and lesions are frustrated by difficulties in identifying ova beyond the genus level. In this study, a newly developed terminal restriction fragment length polymorphism (T-RFLP) method was validated as a tool for differentiating between mixed spirorchiid ova in turtle tissue. Initially, a multiplex PCR was used to differentiate between the five genera of spirorchiid flukes. Following this, PCR was performed using genus/genera-specific fluorescently tagged primer pairs and PCR products digested analysis using restriction endonucleases. Using capillary electrophoresis, this T-RFLP method could differentiate between twelve species and genotypes of spirorchiid flukes in turtles. It was applied to 151 tissue samples and successfully identified the spirorchiid species present. It was found to be more sensitive than visual diagnosis, detecting infections in 28 of 32 tissues that were negative on histology. Spirorchiids were present in 96.7% of tissues tested, with *Neospirorchis* genotype 2 being the most prevalent, present in 93% of samples. Mixed infections were common, being present in 60.7% of samples tested. The method described here is, to our knowledge, the first use of the T-RFLP technique on host tissues or in an animal ecology context, and describes a significant advancement in the clinical capacity to diagnose a common cause of illness in our environment. It is proven as a sensitive, specific and cost-efficient means of identifying spirorchiid flukes and ova in turtles, with the potential to contribute valuable information to epidemiological and pathological studies as well as future diagnostics for this poorly understood disease.

## Introduction

Blood flukes of the family Spirorchiidae are parasites commonly found in endangered green sea turtles (*Chelonia mydas*) from all the major ocean regions [1–3]. They are associated with a range of pathologies, and are thought to be a factor in strandings and mortalities worldwide [4–8]. However, the link between the pathology and the inciting species of spirorchiid has yet to be fully explored. Based on the identification of adult flukes, correlations with gross pathology have been reported. For example, members of the genera *Hapalotrema* and *Learedius* are frequently described from the heart and major arteries, where they are associated with endarteritis, endocarditis, and formation of thrombi [4–6, 8–13]. Species of *Neospirorchis* have been associated with neurological disturbance and disruption of brain tissue integrity [5, 14] although the factors leading to clinical disease have yet to be fully explored. To date, two species of *Neospirorchis* are recognised, and four of *Hapalotrema*. Five species of *Learedius* have been described, however evidence suggests that all are likely to be synonymous with *L*. *learedi* [15]. Three species of *Carettacola*, five of *Amphiorchis* and one species within the genus *Monticellius* are reported from *C*. *mydas* in the literature, though these genera appear to occur at a lower prevalence and their pathogenic affects are less clear.

Spirorchiid ova are often observed histologically in turtle tissues, where they embolise and trigger significant inflammatory responses [4, 5]. Granulomatous inflammation of varying severity is commonly observed in all organs, and may lead to impaired function and potential death of the host [5, 14]. However, the relative pathogenicity of ova of different species is unclear. Spirorchiid ova fall into three broad morphological categories [16] each of which are typical of one or more genera. Identification of ova beyond the genus level is impracticable due to a lack of readily identifiable differentiating features [4, 16]. Molecular methods have the capacity to overcome these limitations and allow further investigation of site tropisms and pathogenicity of individual spirorchiid species.

Terminal restriction fragment length polymorphism (T-RFLP) analysis, a fluorescence based PCR and restriction digestion method, was originally developed to analyse complex microbial communities in environmental samples [17]. Based on the premise of restriction digestion, where enzymes are used to cleave amplicons at polymorphism sites resulting in unique fragment assemblages on gel electrophoresis, T-RFLP presents a number of advantages over traditional PCR-RFLP. The addition of a fluorescent tag on one primer enables the terminal fragment to be detected using capillary electrophoresis, offering precise fragment size determination, increased sensitivity and improved throughput capability [17]. In recent years, the application of this method has expanded into the parasitology field, with T-RFLP assays being developed for *Giardia* [18] and *Cryptosporidium* [19] infections in humans, where they were found to be accurate and efficient in terms of both cost and time.

This paper describes a multiplex PCR T-RFLP method that can be used to accurately identify spirorchiid ova in the tissues of green sea turtles, as it is common for multiple species infections to be present. It is anticipated that the T-RFLP will ultimately assist in investigating the epidemiology and pathology of spirorchiidiasis in green sea turtle populations.

## Materials and Methods

### Collection of reference material

Necropsies were undertaken on dead green sea turtles obtained through wildlife rehabilitation facilities including Underwater World (Mooloolaba) and Australia Zoo (Beerwah) and government agencies (Queensland Parks and Wildlife Service–QPWS) between 2011 and 2014. Adult flukes were collected and identified using morphological and molecular methods as described by Chapman et. al. [15]. Samples of various turtle tissues containing spirorchiid ova were also collected and stored in 70% ethanol at 4°C.

Literature searches were conducted to identify any further described species that may occur in the local area. Reference sequences were obtained from Genbank, and were also provided by the Wildlife and Aquatic Veterinary Disease Laboratory (WAVDL) at the University of Florida.

### Ethics statement

All turtles involved in this study were found dead, died shortly after stranding, or were euthanized on humane grounds after veterinary evaluation at rehabilitation facilities. Euthanasia was performed by veterinarians using intravenous injection of pentobarbital. While no turtles were euthanized specifically for the purposes of this research, all work was completed under approval no. SVS/037/11/ARC/DERM/AUSTZOO issued by the University of Queensland Animal Ethics Committee. Queensland State Government approval to undertake marine research activities was granted through Marine Parks permit no. QS2011/CVL1414 and Scientific Purposes permit no. WISP09021911.

### Extraction of DNA from tissues

DNA was extracted from turtle tissues containing spirorchiid ova using the DNeasy Blood and Tissue kit (Qiagen, Hilden, GER). Procedure was as per the manufacturer’s instructions, except that the samples were homogenised using 1 g of 0.5 mm Zirconia/Silica beads (Daintree Scientific, Tasmania, Australia) in a Biospec Mini-Beadbeater for 3 mins followed by the incubation step. AL buffer (200 μl) was then added to the supernatant and extraction continued as per DNeasy kit instructions. DNA was eluted in 100 μL buffer AE.

### Multiplex PCR amplification

Primers were designed by aligning available spirorchiid 28S rDNA sequences. A specific forward primer was designed for each of *Carettacola* (CarF1) and *Neospirorchis* (NeoF1), as well as a third for *Hapalotrema*, *Learedius* and *Amphiorchis* combined (HapF1). A universal reverse primer (SMR1) was designed, with the resulting amplicons predicted to be of unique sizes distinguishable by gel electrophoresis. Primer details are provided in Table 1.

**Table 1:** Primers designed to target the 28S regions of spirorchiid genera, and 18S Eukaryote primers used as controls to validate DNA quality where negative results for spirorchiids were obtained.

Target genera	Primer (5’– 3’)	Approx. product size	6-FAM tag (2^nd^ round)
*Hapalotrema*	HapF1 CC TTG GGG TTG GTA TGT GTG	443	N
*Learedius*			
*Amphiorchis*			
*Carettacola*	CarF1 TA AGC GTG GTT TGC GCT CG	510	Y
*Neospirorchis*	NeoF1 AG ATC AGT GCA GTG CGT CG	228	Y
Universal reverse	SMR1 GT TAA ACT CCT TGG TCC GTG	-	Y *
Eukaryote Fwd	18SEUDIR TCT GCC CTA TCA ACT TTC GAT GG	140	n/a
Eukaryote Rev	18SEUINV TAA TTT GCG CGC CTG CTG	-	

* denotes 6-FAM tag when paired with HapFI only.

Primer pairs were tested individually against DNA from all available spirorchiid species to ensure specific amplification of a single band of the correct size. To estimate the range of detection of the primer pairs, amplification of 10-fold dilutions of pure genomic DNA from *Hapalotrema*, *Learedius*, *Amphiorchis*, *Carettacola* and *Neospirorchis* was performed. Trials were subsequently run on tissues collected from turtles diagnosed with spirorchiid infection.

PCR was carried out using 200 μM of each dNTP (Bioline–Eveleigh, New South Wales, Australia), 1 x PCR buffer (Qiagen) including 1.5mM MgCl_2_, additional MgCl_2_ to bring the final concentration to 2.5mM, 1 μM of each primer, 1.25 units HotStar Taq (Qiagen) and approximately 100 ng template DNA, with nuclease free water making up a final reaction volume of 25 μL. Cycling conditions comprised an initial activation step of 94°C for 5 minutes, followed by 40 cycles of 94°C for 30 seconds, 57°C for 30 seconds and 72°C for 2 minutes with a final extension step of 72°C for 10 minutes. Five microlitres of PCR product were run in a 2% agarose gel stained using SYBR Safe (Life Technologies Pty Ltd, Grand Island, New York, USA) and visualised using a Bio-rad Gel Doc XR.

Tissue samples returning a negative result for the multiplex PCR were assessed for presence of amplifiable DNA using universal eukaryote 18S gene primers [20] (Table 1). This PCR comprised 100 μM of each dNTP (Bioline), 1 x Green GoTaq Reaction buffer (Promega–Madison, Wisconsin, USA) including 1.5mM MgCl_2_, additional MgCl_2_ to bring the final concentration to 3 mM, 0.4 μM of each primer, 0.5 units GoTaq (Promega) and approximately 100 ng template DNA with nuclease free water making up a final reaction volume of 25 μL. Cycling consisted of an initial activation step of 95°C for 3 minutes, followed by 35 cycles of 94°C for 15 seconds, 60°C for 15 seconds and 72°C for 15 seconds, with a final extension step of 72°C for 2 minutes. Gel electrophoresis was carried out using the same procedure as for the multiplex PCR. Samples that failed to amplify were discarded from the analysis.

### Terminal Restriction Fragment Length Polymorphism

A second round of PCR was performed using single genus-specific primer pairs as indicated by the results of the initial multiplex round. For each reaction, the 5’ end of one primer was labelled with fluorescent 6-FAM dye (Table 2). The PCR reactions and cycling conditions were identical to those described for the first round, except that additional MgCl_2_ was excluded and replaced by an equivalent volume of nuclease-free water.

**Table 2:** Restriction enzymes used to identify each species/genotype following amplification with relevant primer pairs, with predicted 5’ fragment sizes.

	AvaI*	MnlI	BcoDI#	Hpy99I#	AccI	HaeIII
*H*. *postorchis* KM652621.1	443	403	101	443	-	-
*H*. *pambanensis* KM652624.1	442	126	442	442	-	-
*H*. *synorchis* KM652622.1	442	403	443	409	-	-
*H*. *mistroides* KU892016	443	164	443	443	-	-
*L*. *learedi* KM652623.1	359	443	443	443	-	-
*Amphiorchis sp*. KU892017	265	445	445	445	-	-
*C*. *hawaiiensis*/gen 1 KU600068.1–69.1	-	-	-	-	462/463	-
*Carettacola sp*. gen. 2 KU600070.1	-	-	-	-	180	-
*Neospirorchis sp*. gen. 1 KU600072.1	-	-	-	-	-	68
*Neospirorchis sp*. gen. 2 KU600073.1	-	-	-	-	-	158
*Neospirorchis sp*. gen. 3 KU600074.1	-	-	-	-	-	109

* and # denote enzyme pairings for double digests.

The digestion patterns of various restriction endonucleases were predicted using the NEBcutter 2.0 online tool (New England Biosystems, Massachusetts, US). Details of endonucleases used and predicted product sizes are given in Table 2. Reactions were carried out in a total volume of 25 μL, comprising 0.5 μL of restriction endonuclease (New England Biosystems, Massachusetts, US), 2.5 μL CutSmart buffer (New England Biosystems, Massachusetts, US), and 2.5 μL of PCR product with the remainder made up of nuclease free water. Digestions were performed in a thermal cycler for 15 minutes at 37°C. Where possible, endonucleases were combined into a double digest to improve efficiency (Table 2), in which case the reaction contained a total of 1 μL enzyme (0.5 μL x 2), with water volume adjusted accordingly.

To test the ability of the assay to accurately differentiate species in mixed infections, and to determine the lower detection limit, mixtures of DNA were prepared at decreasing ratios for each species and used with the relevant primer pair. Dilutions were made to equal ratios, 1 in 5, 1 in 10 and 1 in 20.

Capillary electrophoresis of digestion products was undertaken by the Animal Genetics Laboratory (AGL) within the School of Veterinary Science, The University of Queensland Gatton Campus. One microliter of sample was diluted 10 fold using nuclease free water and analysed using the Applied Biosystems 3130 Genetic Analyzer. Results were visualised using GeneMapper software (version 5—Applied Biosystems, California, USA)

## Results

Turtles were obtained from various locations on the Queensland coast between Gladstone and Moreton Bay. On the basis of morphology, two species of *Hapalotrema* (*H*. *pambanensis*, *H*. *postorchis*) and one species of *Learedius* (*L*. *learedi*) were identified from examination of 43 *C*. *mydas*. Three distinct genotypes of *Neospirorchis* were detected; these were unable to be characterised morphologically due to difficulty in collecting intact specimens. Three genotypes of *Carettacola* were found, the most prevalent of which was identified as *C*. *hawaiiensis*. *Carettacola* Genotype 1 was only detected from one specimen, and was sufficiently similar to *C*. *hawaiiensis* to be considered a potential variant, whereas Genotype 2 was treated as a separate species, with a restriction endonuclease selected to differentiate it from the former two.

Necropsies of other turtle species indicated the presence of *H*. *synorchis* in the region; while this species has not been reported from *C*. *mydas* to date, it was considered to warrant inclusion in the T-RFLP assay. *Hapalotrema mistroides* and *Amphiorchis sp*., while not reported from the locality to date, were considered possible occurrences and were also included in T-RFLP design. Sequences for *H*. *mistroides* and an unidentified *Amphirorchis* were provided by the WAVDL at the University of Florida, USA. A final genus, *Monticellius*, has not been reported to date from the Pacific Ocean; no material was available for sequencing and no sequences were available in Genbank. Genbank accession numbers for sequences utilised in the assay design are provided in Table 2.

The three primer pairs were initially trialled as single pairs, and subsequently as a multiplex assay. All successfully amplified their respective target templates, and no cross reactivity to non-target genera was observed. Lowest multiplex detection levels achieved from serial dilutions alongside non-target template DNA were 0.11 pg (HapF1/SMR1), 8.70 pg (CarFI/SMR1) and 1.17 pg (NeoF1/SMR1) of DNA.

All enzymes successfully produced fragments of the predicted size when trialled on target amplicons. Proportional dilutions with multiple targets successfully detected DNA down to 1 in 20 in most cases. Template mixes containing *H*. *synorchis* DNA alongside other target species resulted in the HapFI/SMR1 primer pair amplifying *H*. *synorchis* preferentially over other target species. For combinations of other species, both/all species were detectable.

The T-RFLP was successful in diagnosing and characterising spirorchiids from 151 tissue samples collected from 43 central/southern Queensland *C*. *mydas* (Table 3).

**Table 3:** Tissue samples tested by T-RFLP. Summaries of initial visual examination are given for each organ, including gross necropsy and histology findings. Columns headed with spirorchiid species/genotype names summarise T-RFLP results for each organ. Columns labelled ‘PCR only’ refer to positive PCR results where no subsequent T-RFLP result could be obtained.

	Bladder	Brain	FP	Gall bladder	G.I. tract	Heart	Kidney	Liver	Lung	Ovary	Pancreas	Spleen	Testis	Thrombus	Thyroid	Total
No. samples	2	43	2	2	18	12	14	11	14	1	8	12	2	3	7	151
No. examined -histology	0	31	1	0	8	9	9	8	10	0	5	7	0	3	6	97
Ova observed—histology	-	25	1	-	3	4	7	4	7	-	4	6	-	3	1	65
Ova observed—gross	0	8	0	0	9	0	0	0	2	0	0	0	0	0	0	19
Adult observed—gross	0	1	0	0	0	0	0	0	0	0	0	0	0	0	0	1
*Hapalotrema postorchis*	0	5	0	1	6	3	3	4	3	0	4	6	0	1	2	38
*Hapalotrema pambanensis*	1	8	1	1	5	4	2	3	3	0	4	7	0	2	2	43
*Hapalotrema synorchis*	0	0	0	0	0	0	0	0	0	0	0	0	0	0	0	0
*Hapalotrema mistroides*	0	0	0	1	0	0	0	1	3	0	0	3	0	0	2	10
*Learedius learedi*	0	6	1	1	1	2	1	1	3	0	2	2	0	0	0	20
*Amphiorchis sp*.	0	0	0	0	0	0	0	0	0	0	0	0	0	0	0	0
*Hapalotrema sp*. (PCR only)*	0	1	0	0	1	0	0	0	0	0	0	0	0	0	0	2
*Carettacola hawaiiensis* / gen. 1	0	2	0	0	2	0	0	2	0	0	0	1	0	0	0	7
*Carettacola* gen. 2	0	0	0	0	0	0	0	0	0	0	0	0	0	0	0	0
*Carettacola sp*. (PCR only)	0	2	0	0	0	0	0	0	0	0	0	0	0	0	0	2
*Neospirorchis* gen. 1	0	13	1	0	3	5	6	2	4	0	4	3	1	1	3	46
*Neospirorchis* gen. 2	2	40	2	1	15	10	14	9	13	1	8	12	2	3	7	139
*Neospirorchis* gen. 3	0	0	0	0	1	0	3	0	6	0	0	1	0	0	0	11
*Neospirorchis sp*. (PCR only)	0	0	0	0	1	0	0	0	0	0	0	0	0	0	0	1
No spirorchiids detected	0	1	0	0	1	0	0	2	1	0	0	0	0	0	0	5

* refers to all genera within the *Hapalotrema/Learedius/Amphiorchis* group as targeted by the HapF1/SMR1 primer pair. G.I. = gastrointestinal, FP = fibropapilloma.

A total of 153 samples were tested, with seven tissues failing to amplify any spirorchiid DNA on initial multiplex PCR. Of these, five samples which showed positive amplification of eukaryote 18S gene, indicating the integrity of DNA were therefore determined to be valid negative results. The results from the remaining two samples were discarded due to apparent poor DNA quality as shown by negative PCR results to eukaryote 18S gene. In total, only 3.3% of tissue samples tested returned validated negative results, indicating a high prevalence of spirorchiid infection across all turtles and tissues tested.

Of 97 tissues that were examined histologically, spirorchiid ova were observed in 65. All of these tissues also returned positive T-RFLP results. Thirty-two further tissues showed no histological evidence of spirorchiids; of these, only four returned a validated negative result on T-RFLP. Further, adult flukes were obtained from only two tissues during gross necropsy. Both tissues returned positive T-RFLP results for the relevant species.

Ninety-one samples (60.7%) were found to have mixed infections of two or more spirorchiid species/genotypes. Most target genera and species were detected, with the exceptions of *H*. *synorchis*, *Amphiorchis sp*., and *Carettacola* genotype 2. The most often detected species was *Neospirorchis* genotype 2, which was present in 93.3% of samples tested, and found in all tissue types. *Neospirorchis* genotypes 1 and 3 were observed at lower frequencies (30.7% and 7.3% respectively). *Neospirorchis* genotype 1 was also found in nearly all tissue types, while the third genotype was restricted to the gastrointestinal tract, kidneys, lung and spleen. *Carettacola* spp. occurred at a low prevalence and were detected in 9 of 151 samples (6%). Tissues returning positive results included the liver, gastrointestinal tract, spleen and brain. Of the *Hapalotrema/Learedius/Amphiorchis* group, *H*. *postorchis* (24.5%) and *H*. *pambanensis* (28.5%) were most common. *Learedius learedi* was detected regularly, being present in 13.25% of samples. All three species were found across most tissue types. *Hapalotrema mistroides* was less common (6.6%) and appeared to have a more restricted distribution, only being noted in lung, spleen, thyroid and gall bladder samples. *Hapalotrema synorchis* and *Amphiorchis sp*. were not detected in any tissue. A more detailed analysis of relative prevalence, tissue tropisms and pathology associated with each species/genotype will be described in a subsequent publication.

## Discussion

The T-RFLP method described herein has proven a cost-effective method for identifying spirorchiid ova in the tissues of *C*. *mydas*. Insufficient intra-genus variation exists within common sequencing regions for the Spirorchiidae (e.g. internal transcribed spacer 2, 28S) to allow the design of species-specific primers. In lieu of these, methods such as RFLP provide a simple and accessible alternative to resolving identities of spirorchiid ova where mixed infections are likely. To our knowledge, this is the first veterinary application of T-RFLP methodology, and the first such assay designed to identify parasites directly in host tissue.

Traditionally, gross necropsy and histology have been the gold standard in spirorchiid detection, however these methods have significant limitations. Spirorchiid ova are frequently not grossly observable in tissues except in cases of heavy deposition or granuloma formation. Some adult flukes are microscopic and become tightly lodged in small blood vessels, making them difficult to detect and recover [4, 6]. Molecular approaches are not affected by these problems and therefore provide substantial advantages over visual detection methods. This assay was able to detect spirorchiids in 100% of histologically confirmed infected tissues, but, significantly, it was also able to detect infections in 28 of 32 tissues in which ova were not visually observed.

This assay’s ability to differentiate between spirorchiid ova also presents many advantages to those seeking to understand spirorchiidiasis. Foremost, specific identification of ova allows correlations to be drawn between spirorchiid species and pathology. This information opens opportunities for targeted diagnostics; as spirorchiid infection is almost universal among marine turtles across the globe [4–6], a generic spirorchiid diagnosis is generally of limited value. Additionally, the specific data obtained provides a cheap and rapid means to gain insights into geographic prevalence patterns among taxa, and also into broad and fine scale tissue predilections within the host.

Capillary electrophoresis facilities are commonplace in molecular service laboratories, and are therefore readily accessible in the majority of institutions. This method has the advantage of greater fragment resolution accuracy over gel electrophoresis, and can differentiate between fragments varying by only a few base pairs in size [21]. In addition, sensitivity and separation time is improved [21] compared with gel-based visualisation. Capillary electrophoresis therefore represents a cost and time efficient option for the visualisation of restriction products.

In this study, *H*. *synorchis* was found to amplify preferentially over other species targeted by the HapFI/SMR1 primer set. Selective amplification by primers is a recognised problem when handling mixed templates, and indeed was observed by Waldron et al. [19] who found that *Cryptosporidum parvum* was preferentially amplified over *Cryptosporidium hominis*. *Hapalotrema synorchis* has not previously been reported from *C*. *mydas*, and was not detected during these trials on turtle tissue samples, despite 151 tissues from 43 turtles being tested. Thus, this particular issue does not present a problem in the use of this assay for the diagnosis of spirorchiidais in *C*. *mydas*, the most frequently studied marine spirorchiid host. However, care should be taken in interpretation of results if applied to other host species where *H*. *synorchis* is well documented i.e. *Eretmochelys imbricata* and *Caretta caretta* [2, 22].

The species presence and prevalence data presented here are generally in accordance with previously published observations. As discussed, adult *H*, *synorchis* has not been reported from *C*. *mydas*, and no evidence to the contrary was found in this ova focussed study. *Amphiorchis* sp. has not been reported from the western Pacific Ocean to date, and no evidence of its presence was found here. The sequence used in the design of this study was from a specimen collected from *C*. *caretta*, however, *Amphiorchi*s sp. are reported from *C*. *mydas* and spirorchiids within the *Hapalotrema/Learedius/Amphiorchis* group often infect multiple host species around the globe [2]. *Hapalotrema mistroides* has not been recorded from Australia’s east coast previously, nor has it been reported from the Pacific Ocean. This study detected *H*. *mistroides* ova in turtles from central and southern Queensland, representing an expansion in the known range of this species. *Carettacola* sp. were found at a low prevalence (6%). While the lower DNA detection limit of the CarFI/SMR1 primer pair was slightly higher than observed for other primer pairs, this relatively low occurrence is reflective of observations made by Work et al. [23], who noted that *C*. *hawaiiensis* were less predominant than species of *Hapalotrema* or *Learedius* in Hawaiian *C*. *mydas*. The very high prevalence (93%) of *Neospirorchis*, while significant, is not unprecedented in the locality. Gordon et al. [6] reported that 98% of turtles in their turtle mortality study showed signs of spirorchiid infection, based either on the presence of adult flukes, characteristic lesions or histological detection of ova. Microscopic flukes were observed histologically in 72% of turtle brains; samples were collected from two turtles and identified as *N*. *schistsomatoides*. Their study did not attempt to identify eggs present in histological sections, noting the difficulties in distinguishing ova often presented in cross section and in a distorted state.

To date, the diversity and phylogeny of the spirorchiids has not been fully explored; in particular, genera such as *Neospirorchis*, *Amphiorchis* and *Monticellius* are poorly understood and little genetic data is available for them. As more information becomes available, the opportunity will arise to further refine molecular identification assays. Regardless, the method described here has the capacity to provide valuable data contributing to the conservation of threatened *C*. *mydas* populations, and those of other sea turtles.
